# High-Throughput Field Plant Phenotyping: A Self-Supervised Sequential CNN Method to Segment Overlapping Plants

**DOI:** 10.34133/plantphenomics.0052

**Published:** 2023-05-18

**Authors:** Xingche Guo, Yumou Qiu, Dan Nettleton, Patrick S. Schnable

**Affiliations:** ^1^ Department of Statistics, Iowa State University, Ames, IA, USA.; ^2^ Plant Sciences Institute, Iowa State University, Ames, IA, USA.; ^3^ Department of Agronomy, Iowa State University, Ames, IA, USA.

## Abstract

High-throughput plant phenotyping—the use of imaging and remote sensing to record plant growth dynamics—is becoming more widely used. The first step in this process is typically plant segmentation, which requires a well-labeled training dataset to enable accurate segmentation of overlapping plants. However, preparing such training data is both time and labor intensive. To solve this problem, we propose a plant image processing pipeline using a self-supervised sequential convolutional neural network method for in-field phenotyping systems. This first step uses plant pixels from greenhouse images to segment nonoverlapping in-field plants in an early growth stage and then applies the segmentation results from those early-stage images as training data for the separation of plants at later growth stages. The proposed pipeline is efficient and self-supervising in the sense that no human-labeled data are needed. We then combine this approach with functional principal components analysis to reveal the relationship between the growth dynamics of plants and genotypes. We show that the proposed pipeline can accurately separate the pixels of foreground plants and estimate their heights when foreground and background plants overlap and can thus be used to efficiently assess the impact of treatments and genotypes on plant growth in a field environment by computer vision techniques. This approach should be useful for answering important scientific questions in the area of high-throughput phenotyping.

## Introduction

High-throughput phenotyping enables large-scale collection of plant images and sensor data in greenhouses and fields [1–7]. For example, side-view images can be taken of hundreds to thousands of crops in fields simultaneously and continuously throughout their growth period by placing cameras in front of each row of plants [8]. To utilize these rich data for statistical analyses of plant phenotypes, subsequent image processing for trait extraction is required.

Plant object segmentation is a fundamental step in plant trait extraction [9,10]. There are many existing plant image processing tools, such as Scanalyzer [11], CropSight [12], and Leaf-GP [13]. These tools typically apply a simple thresholding method [14,15] for plant segmentation, which works well for greenhouse images with homogeneous backgrounds [16,17]. However, it fails to produce satisfactory plant segmentation results for images with complex backgrounds, especially in-field plant images. Neural network methods, such as U-net [18] and SoySegNet [19], can more accurately segment plant images with noisy backgrounds. However, these methods are based on supervised learning with human-labeled data or semisupervised learning with a mixture of human-labeled data and unsupervised data. Preparing a sufficiently large training dataset is both time consuming and labor intensive. Guo et al. [8] and Adams et al. [20] proposed 2-step self-supervised methods of image segmentation, in which training data (labels of plant pixels) are first produced by *K*-means clustering of greenhouse images with a clean background, and then neural network models are trained, based on these automatically generated training data, to perform segmentation of both greenhouse and field-grown plants. They demonstrated that their methods are more accurate and robust than the traditional thresholding methods.

**Fig. 1.**
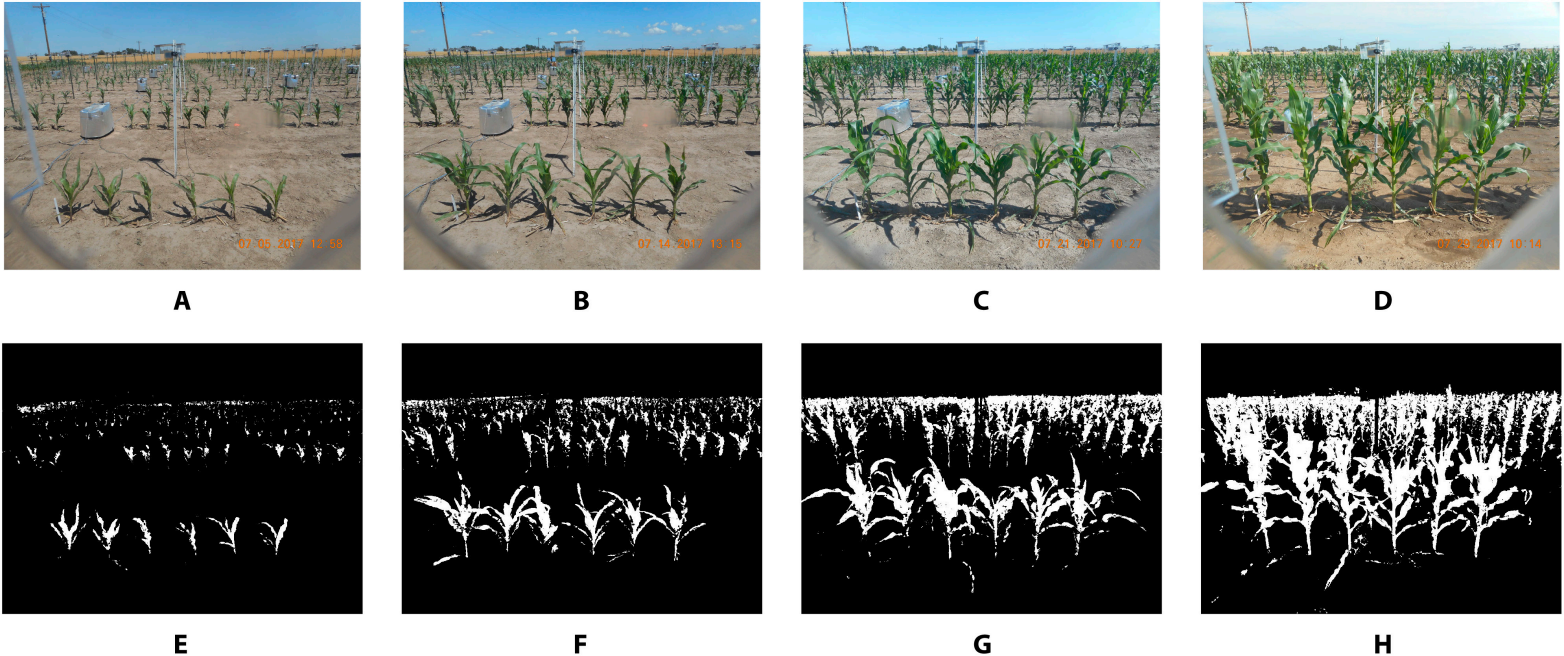
Segmentation of images taken over the plant growth period. (A to D) Sequence of plant photos taken by one camera over time in one row of a field phenotyping system at Iowa State University. (E to H) Corresponding images segmented using the KAT4IA procedure from [8].

Plant segmentation is especially challenging for field images because the background is composed of a mixture of dirt, equipment, plant shadows, etc. Figure 1A to D shows a sequence of plant photos taken by a single camera over time in one row of the field phenotyping system at Iowa State University. Not only is there a complex background, but also, as plants grow, the target plants in the foreground row overlap with the background plants, so it is difficult to separate the rows of plants even with the human eye. The *K*-means assisted training for image analysis (KAT4IA) procedure proposed by Guo et al. [8] can produce well-segmented images, removing most background noise (see the segmented results in Fig. 1E to H). Based on the segmented images, KAT4IA can provide valid height measurements for plants in early growth stages, when the plants in the foreground row do not overlap with those in the background, as in Fig. 1E and F. However, KAT4IA cannot separate the target plants after they overlap with the background plants (see Fig. 1H as an example). Therefore, although the existing methods can accurately estimate in-field plant heights during early growth stages, they fail when plants overlap and cannot provide complete growth curve estimates. Because of space limitations in experimental fields, the distance between plant rows often needs to be small; thus, in many experiments, plants quickly begin to overlap as they grow. It is therefore important to find an automated machine learning method to separate overlapping plants that does not require human-labeled training data.

To solve this problem, we propose a self-supervised sequential convolutional neural network (SS-CNN) to separate foreground-plant pixels from background-plant pixels. We construct a computational pipeline to extract plant height data and estimate the entire growth curve of each separated plant. The key idea is to use the segmentation results from the images before plants overlap as the training data for the images in which plants overlap, in a sequential way over the course of plant growth. Our strategy relies on the assumption that the pixel intensities of the foreground plants before the foreground and background plants overlap are sufficiently similar to those of foreground plants after the overlap but before the physiological maturity stage of maize when the plants begin to turn yellow.

Specifically, we first use greenhouse images to train a plant segmentation method for field-grown plants as proposed by Guo et al. [8]; the method aims to segment all plant pixels and remove nonplant background noise. Then, using the proportion of plant pixels in each early-season image row, we identify the foreground-plant and background-plant pixels from images with nonoverlapping plants. In this way, self-supervised training data about plant pixels can be automatically and efficiently constructed and used for the separation of overlapping plants in the late growth stage. This self-supervised method avoids the expensive manual labeling process of preparing training data.

Distinguishing between foreground-plant and background-plant pixels is more challenging than distinguishing between plant and background pixels. Therefore, neighborhood information for each pixel is needed to obtain a good classification result. We construct a convolutional neural network (CNN) model based on the pixel intensities from a rectangular neighborhood, which uses the geometric structure of plants to better separate the foreground-plant and background-plant pixels. Plant heights can then be measured based on the segmented foreground-plant images. By combining the late-growth-stage height measurements from the proposed method and the early-growth-stage height measurements computed by the KAT4IA procedure, the complete growth curves of plants throughout their growth periods can be obtained. The extracted growth curves can be used for subsequent biological analysis. In Results, we present the results from functional principal components analysis (FPCA) [21,22] to study and compare the growth dynamics of different genotypes based on extracted heights, revealing the impact of genotypes on plant growth patterns.

## Methods

Our primary goal is to develop a pipeline to automatically separate foreground-plant pixels from background-plant pixels, as shown in Fig. 1, and extract the heights of all foreground plants from a photo sequence to estimate plant growth curves. The main steps are as follows. Detailed procedures for each step are explained in the subsequent subsections.

1. Segmentation from background: The KAT4IA algorithm [8] was performed starting with the sequence of field images taken by a camera over time to obtain segmented images of plants. The background from images was removed by replacing the RGB intensities of all the classified nonplant pixels with a 0. This will keep the original RGB intensities for the plant pixels and color all background pixels black; the resulting images are known as “background-removed images.” The time point was identified when foreground plants started to overlap background plants.

2. Automatic construction of training data for plant separation: The foreground and background plants were separated in the background-removed images before the plants overlap. The training data were created by labeling all foreground- and background-plant pixels with 1 and 0, respectively. For each labeled pixel, the pixel intensities were included in its neighborhood with size (2*r* + 1) × (2*c* + 1) as the associated features for this pixel, where *r* and *c* are the half width and half height of the neighborhood rectangle centered on the labeled pixel.

3. CNN learning: A CNN was trained using the training data obtained from the second step to separate the foreground and background plants in later-stage images, where foreground plants and background plants overlap.

4. Postprocessing of classification results by superpixels: The classification results of the foreground- and background-plant pixels were refined from the third step using simple linear iterative clustering (SLIC) superpixels proposed by Achanta et al. [23]. A common label was assigned to all pixels within each superpixel.

5. Height measurements: The plant height was calculated for each foreground plant from a sequence of images over the growing season. The plant growth curve was estimated using nonparametric regression with a nondecreasing mean [24,25].

All steps are implemented by *R language* and the API *Keras* in *R*. The training model was built on a single personal computer, and the segmentation of the field images based on the trained model was performed in high-performance computing clusters with parallel computing.

### Image data and plant segmentation from background

For this project, we use field images taken in a rainfed (i.e., nonirrigated) field near Grant, Nebraska at GPS coordinates (40.94, −101.77) in 2017 by researchers from the Plant Science Institute of Iowa State University. The field contains 2 replicate plantings, with 103 and 101 genotypes of maize, respectively. Each row in each replication includes up to 6 plants of a single genotype, and one camera is installed in front of each row of plants. The row length, between-row space, and within-row plant space were 182.9, 304.8, and 36.6 cm, respectively. We used NIKON COOLPIX S3700 cameras in front of each row of plants with a focal length of 4.5 mm and a sensor size of 6.17 mm × 4.55 mm. The camera to row distance was at 213.4 cm, and the height of the camera was roughly about 120 to 150 cm mounted on top of each pole. The average number of photos taken per camera is 1,719 and 1,650 for the 2 replications, respectively, with photos taken every 15 min during daylight hours. We use photos taken from mid June to early August. The field photos are RGB images with intensity values of red, green, and blue channels between 0 and 255 for each pixel. Pixel intensities are normalized by dividing them by 255, producing floating point numbers between 0 and 1. We rescale the image resolution from 5,152 × 3,864 to 1,000 × 750 to increase computation efficiency.

**Fig. 2.**
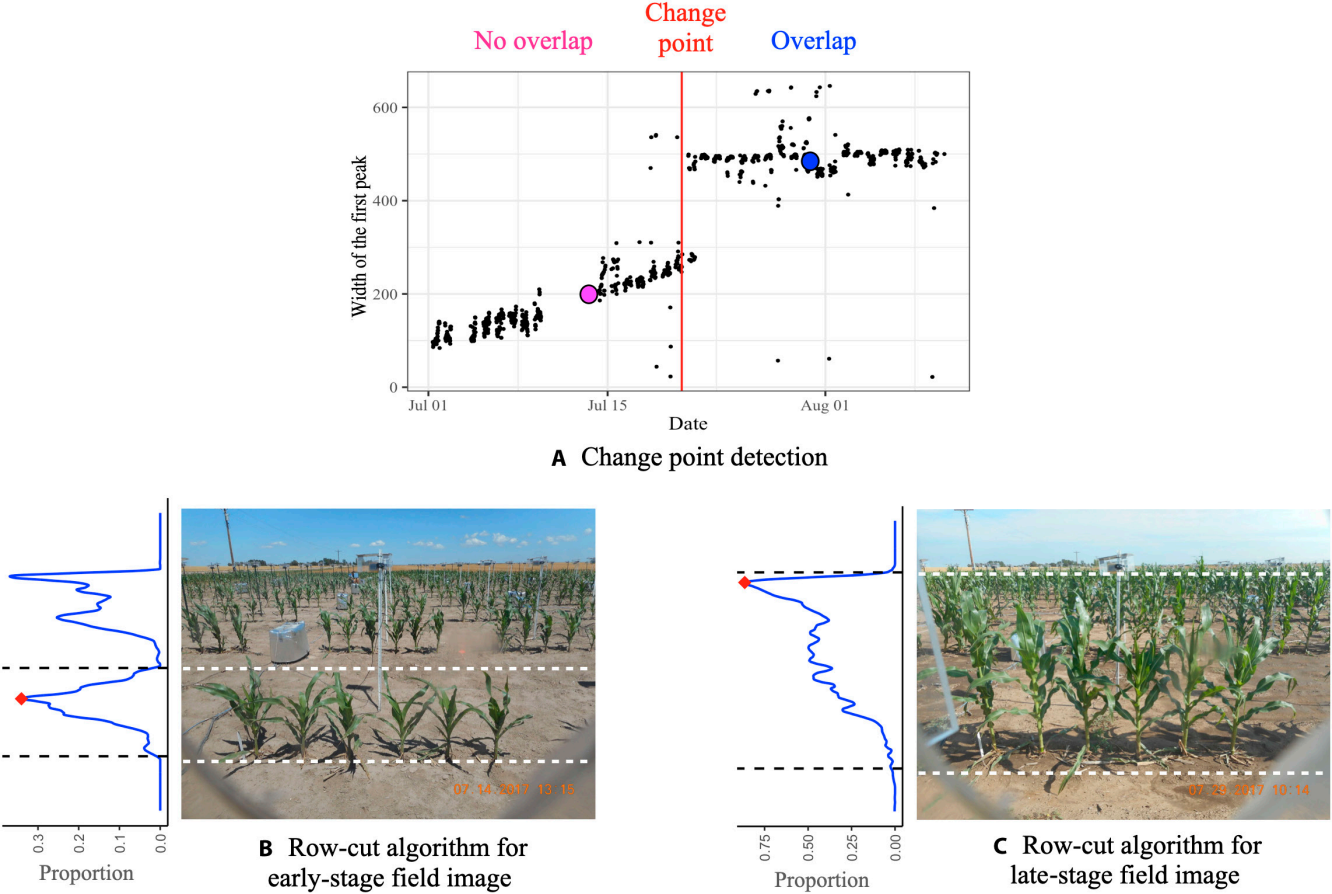
Example of how the row-cut algorithm separates foreground plants. (A) Scatterplot of the width of the identified first peak from the sequence of images over time. The red line is the time point when the foreground and background plants start to overlap (“change point”). (B) Result of the row-cut algorithm for an early-growth-stage field image; the pink dot in (A) indicates the estimated width of the first peak in (B). The row proportion curve of the segmented image is shown on the left-hand side of the field image. The 2 dashed lines are the upper and lower boundaries of the first peak from the bottom. (C) Result of the row-cut algorithm for a late-growth-stage field image; the blue dot in (A) indicates the estimated width of the first peak in (C).

**Fig. 3.**
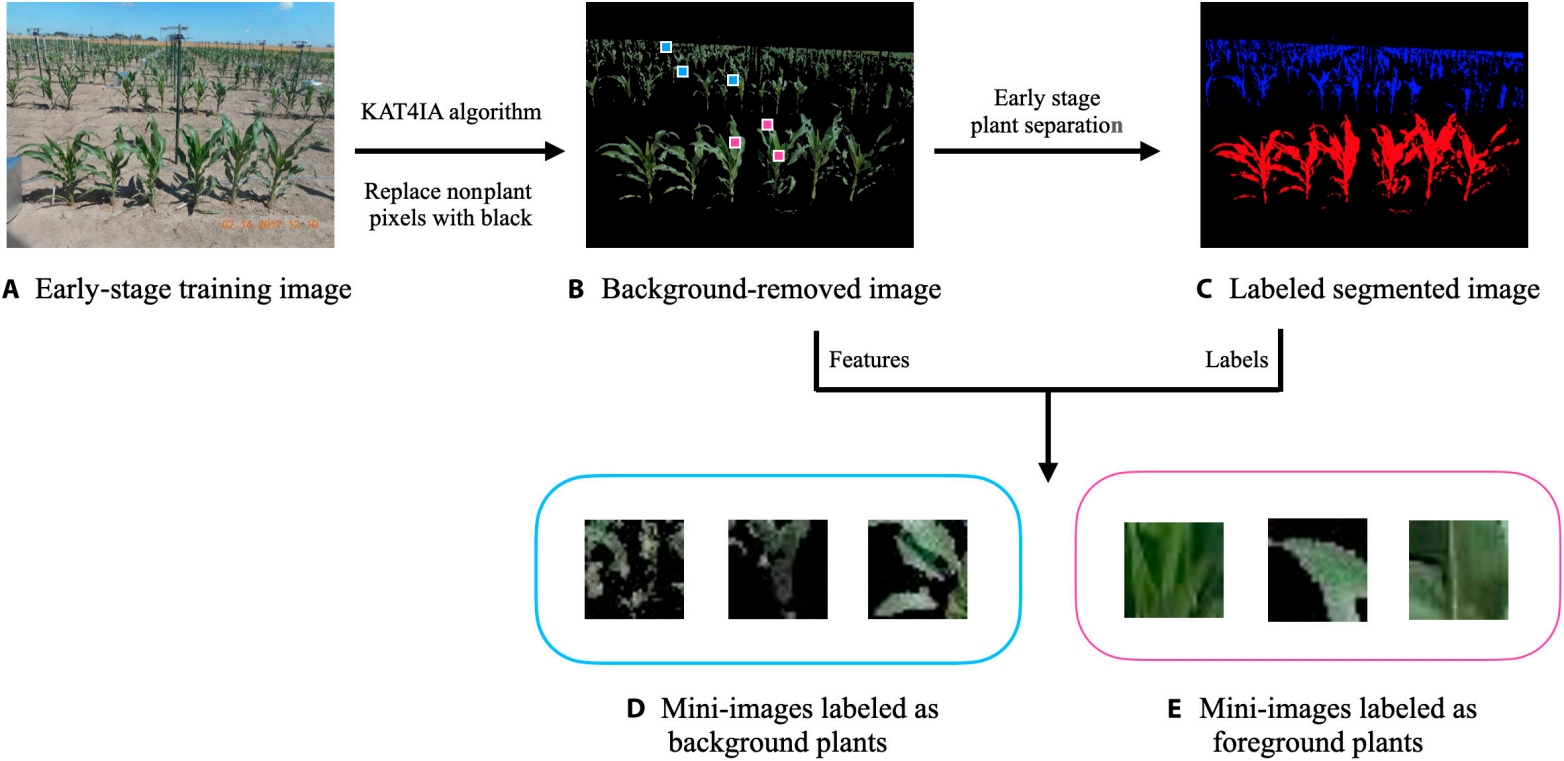
Workflow for constructing a training dataset. (A) Example early-growth-stage field image. Applying the KAT4IA algorithm to (A) gives the “background-removed image” (B). Separating foreground and background plants using the row-cut algorithm gives the “labeled segmented image” (C). (D and E) Cropped mini-images from 3 example background-plant pixels marked in (B) using blue rectangles and 3 example foreground-plant pixels marked in (B) using pink rectangles.

In our training pipeline, we first apply the KAT4IA procedure proposed by Guo et al. [8] to obtain segmented images of plants for in-field images (Fig. 1). KAT4IA uses greenhouse plant images to train the in-field segmentation model (see [8] for more details). Using the segmentation results, we then remove the background from the images by replacing the RGB intensities of all nonplant pixels with zero values (Fig. 3B). This resulted in “background-removed images.” For each row of plants, we use the following method to automatically detect the time point when the foreground and background plants overlap. First, the proportion of plant pixels in each image row is calculated. Then, the first peaks (from the bottom to the top of an image) in the row proportions are identified by choosing the upper and lower boundaries of each peak as the first pixel rows smaller than a small percentage (e.g., 5%) of the peak maximum above and below the center of the peak. The boundaries of the first peak of the row proportions identify the image rows with the foreground plants. The row-cut algorithm mentioned above helps locate foreground plants (see Fig. 2B). A change-point detection method is then applied to the width of the identified first peak from the sequence of images over time to estimate the time that foreground and background plants overlap for each row in the field (Fig. 2A).

With this approach, the width of the first peak will increase sharply to a larger value when the plants begin to overlap. The row-cut algorithm can accurately identify the image rows of the foreground plants in the early growth stage, when the foreground and background plants are well separated. However, once foreground plants begin to overlap with background plants, the row-cut algorithm tends to segment the image rows containing both foreground and background plants, which results in a jump in the width of the segmented rows (see example in Fig. 2C). This jump corresponds to the date when the foreground plants begin to overlap with the plants in the background. Figure 1C provides an example.

### Automatic construction of training data

To separate overlapping foreground and background plants, a large set of training data is needed to build machine learning algorithms. However, obtaining such training data usually requires manual annotation and labeling on each plant pixel in a large set of images for every experiment. This labeling process is both time consuming and labor intensive due to the high resolution of the images and the irregular shape of plants. We address this challenge by using the plant pixels from the nonoverlapping plants in the early growth stage to construct self-supervisory training data for separating overlapping foreground and background plants in the late growth stage. Specifically, for early-growth-stage in-field images, foreground and background plants can be simply separated using the row-cut algorithm in the previous step, as there is a sharp valley in the curve of the row proportions of the segmented image if the foreground and background plants do not overlap. Thus, we can have the algorithm simply define all plant pixels above a cutoff line as background-plant pixels (0) and all plant pixels below the cutoff line as foreground-plant pixels (1) (see example in Fig. 3C).

Moreover, to construct more representative training data of plants in late growth stages, we use the images from right before the overlap begins. For example, Fig. 1C is better suited for constructing training data than Fig. 1A and B because the plant structure in Fig. 1C shares more similar characteristics to plants in the late growth stage. As the neighborhood pixels contain geometric information about the plants, they can help to distinguish the target plants. For each labeled pixel of foreground and background plants, the cropped mini-image centered at that pixel with neighborhood (2*r* + 1) × (2*c* + 1) is used as the input features, where *r* and *c* are the half width and half height of the neighborhood rectangle centered on the labeled pixel. The intuitive presumption is that cropped mini-images within the same category are more likely to share similar characteristics. This is similar to classical CNN approaches in which convolution of neighborhood information is used to predict the response category. The workflow for constructing a training dataset is summarized in Fig. 3. Examples of background and foreground-plant features are shown in Fig. 3D and E, respectively.

### Separation of foreground- and background-plant pixels by CNN

Next, we use the training data generated from the images before plants overlapped to train a CNN to separate overlapping foreground and background plants, using the API *Keras* in *R*. For each labeled pixel in the training data, the RGB (red, green, and blue) intensities of the pixels in its (2*r* + 1) × (2*c* + 1) neighborhood are used as the input features, where *r* = *c* = 16. This results in a feature space with dimensions 33 × 33 × 3 for each training pixel, where 3 is the number of channels for red, green, and blue intensities, and 33 × 33 is the resolution for each channel. We also tried the CNN models with *r* = *c* = 8, *r* = *c* = 12, and *r* = *c* = 20. From the training results, we found that the validation accuracy increased with the increase of *r* and *c*, and became stable after *r* and *c* reached 16. Therefore, we chose the neighborhood size as *r* = *c* = 16, since a smaller neighborhood size leads to less training time.

**Fig. 4.**
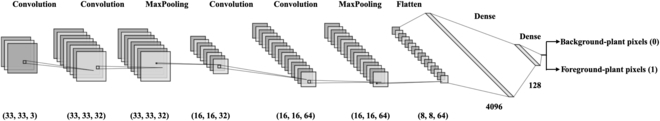
The structure of the CNN for classifying foreground- and background-plant pixels for the images with overlapping plants in the late growth stage.

Specifically, in the CNN model, there are 4 convolution layers with the following sizes: (33,33,32), (33,33,32), (16,16,64), and (16,16,64). The first of these is the input layer, where 3 is the number of channels, and 33 × 33 is the resolution for each channel. A 3 × 3 filter kernel with the Same Padding and the ReLU activation function is used to calculate the convolution layers. A 2 × 2 max pooling with no padding is applied between the second and third convolution layers. Another 2 × 2 max pooling is applied after the last convolution layer, which results in a max pooling layer with size (8, 8, 64). Finally, a multilayer perceptron (MLP) with one hidden layer is used to compute the predicted probability of a particular pixel belonging to the foreground-plant class. Flattening the max pooling layer gives the input layer of the MLP, which has 4,096 nodes. The hidden layer has 128 neurons. The dropout rates between the input layer and the hidden layer of MLP, and between the hidden layer and the output layer, are set at 0.3. The ReLU activation function is used between the input layer and the hidden layer of the MLP. The sigmoid activation function is used to predict the foreground probability of each pixel based on the hidden layer of the MLP. The binary cross-entropy loss function with the Adam optimization algorithm and a learning rate of 0.001 is used to evaluate the network model. Finally, 100 epochs with a batch size of 1,000 are used for the training, and 5% of the training data are retained as the validation set. The average training and validation accuracy of the last 50 epochs are 97.7% and 94.3%, respectively, indicating a good fitting result for the plant CNN. Figure 4 is the diagram that shows the structure of the proposed CNN.

**Fig. 5.**
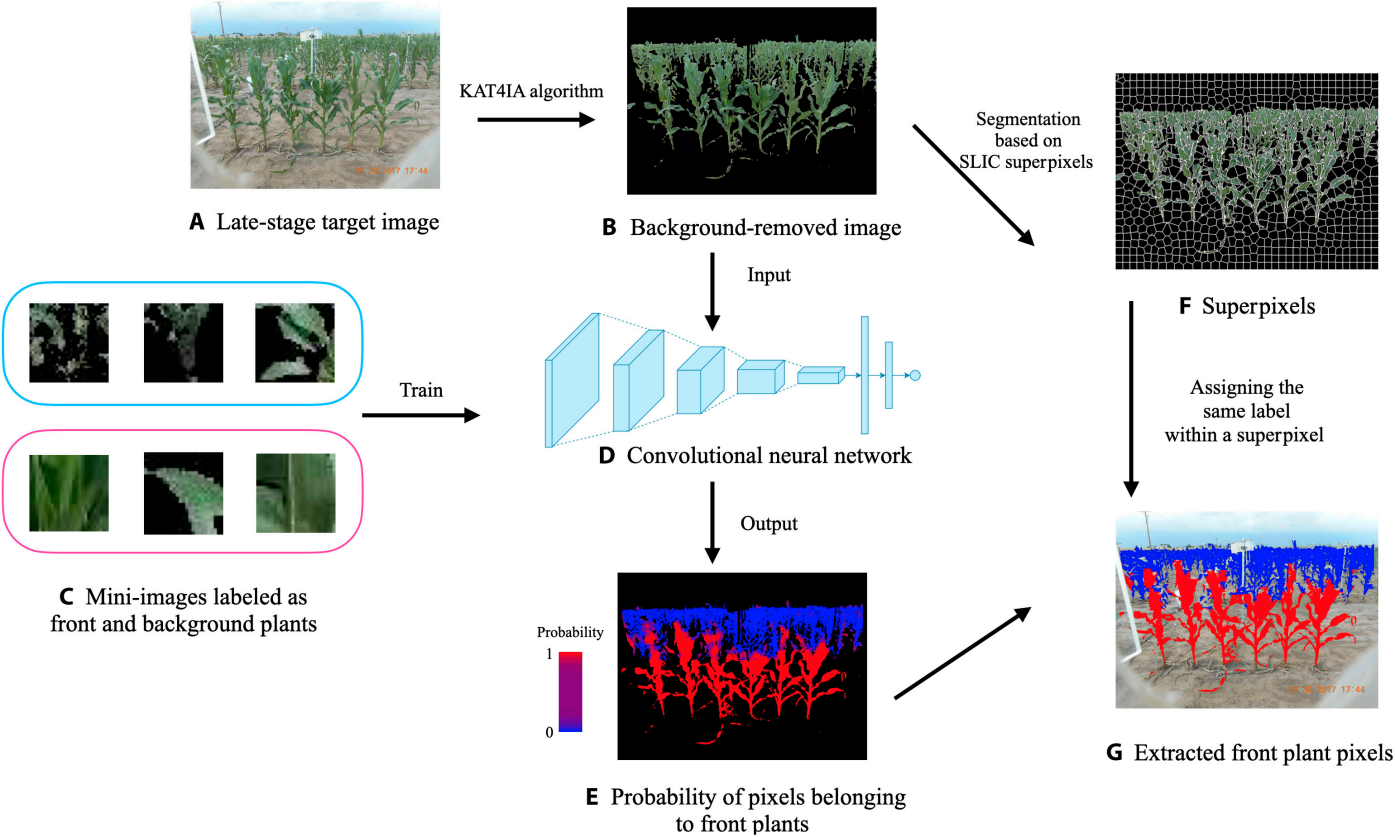
Plant pixel separation and postprocessing workflow. (A) Target late-growth-stage field image. The goal is to extract the foreground plants from the image. Applying the KAT4IA algorithm to (A) gives the “background-removed image” (B). The superpixels of (B) are shown in (F). Cropped early-growth-stage mini-images associated with 2 plant classes are collected as training data (C) to train the CNN (D). For each plant pixel in (B), the neighborhood mini-image is cropped, and then applying (D) to each mini-image gives the probability of each pixel belonging to the foreground-plant class. The probabilities are visualized in (E), where red indicates that a 1 was assigned and blue indicates that a 0 was assigned. The final image (G) is obtained by first averaging the predicted probability within each superpixel and then classifying the foreground-plant and background-plant superpixels using a cutoff threshold value.

The CNN is trained to classify the foreground- and background-plant pixels for the images with overlapping plants in the late growth stage, and the probability of each plant pixel belonging to the foreground-plant class is computed. Figure 5E shows the predicted probabilities from an example image.

### Postprocessing of CNN results using superpixels

The proposed self-supervised method classifies each plant pixel in the background-removed image based on the probabilities generated from the CNN model using a given cutoff value. Figure 5E shows that our trained CNN separates foreground plants from background plants reasonably well. However, there are still some classification errors: The image in Fig. 5E includes several red points (identified foreground pixels) among the blue points (identified background pixels) and several blue points surrounded by red points. Note that the CNN predicts the class label for each pixel separately. This may lead to a nonsmooth segmentation result for the target plants. Therefore, we considered that it should be possible to refine the classification results from the CNN by utilizing the image geometry information, for example, the surrounding pixel colors and the spatial proximity of pixels.

To this end, we incorporated into the pipeline a process in which SLIC superpixels [23] are formed on the background-removed images to group pixels into perceptually meaningful atomic regions based on pixel coordinates and RGB intensities (e.g., Fig. 5F). The superpixels can be interpreted as geometric miniclusters of each image that share similar information. To borrow information from neighboring pixels, the average probability of the foreground class within each superpixel is calculated. A cutoff threshold of 0.5 is then used to classify the foreground and background plants at the superpixel level. All the pixels within a superpixel are predicted to be of either foreground or background class if the average probability is larger or smaller than 0.5, respectively. In this way, all pixels in the same superpixel are classified into a common class. To accelerate the computation, the average probability of the foreground class in the *i*th superpixel is estimated based on a random sample of $N_{i}^{\alpha }$ pixels from this superpixel, where *N_i_* is the total number of pixels in the *i*th superpixel. Figure 5 summarizes the workflow for plant pixel separation using the self-supervised CNN and postprocessing using superpixels, which identifies foreground plants with high accuracy.

### Plant height measurement and growth curve fitting

For early-growth-stage images, before plants overlap, foreground plants can be easily separated using the row-cut algorithm (Fig. 2). Individual foreground plants are then separated using a column-cut algorithm similar to the row-cut algorithm. Then, the height of each plant is measured based on the top and bottom plant pixels in the segmented image. This process for early-stage plants is illustrated in detail in the KAT4IA pipeline [8].

**Fig. 6.**
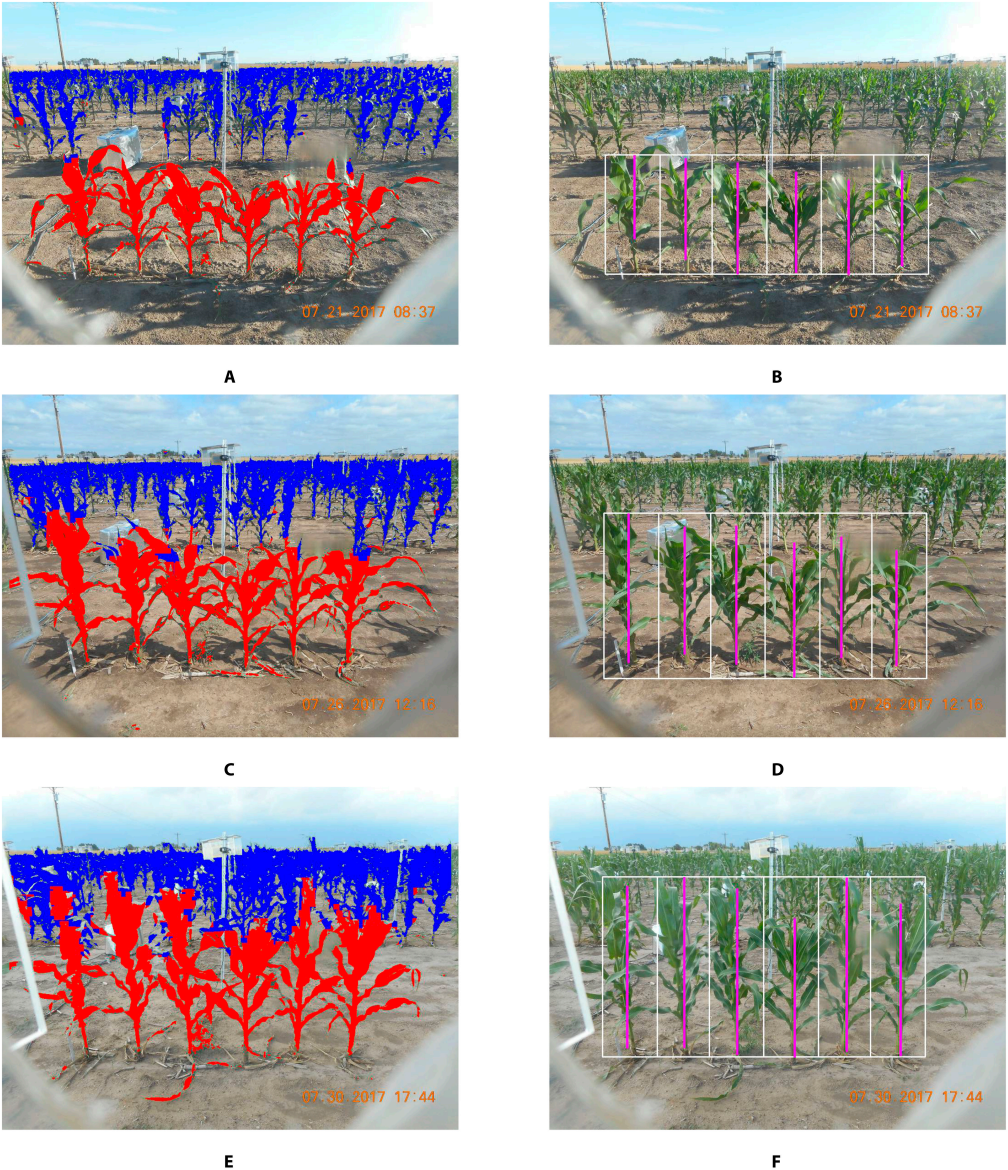
Foreground plants are identified, and their heights are determined. (A, C, and E) Foreground- and background-plant identification results for 3 example images from one camera. (B, D, and F) Corresponding height measurement results.

For late-growth-stage images, once the foreground plants are identified and separated, a similar height measurement algorithm can be used. First, a binary image is created in which foreground-plant pixels are labeled 1 and all other pixels are labeled 0. Row means are then calculated, giving the percentage of foreground-plant pixels in each row of the image. Second, the row mean curve is smoothed using a local smoother using the *loess* function in *R*. The maximum of the row means is calculated, and the upper and lower boundaries of the peak in the row mean curve are identified as the first rows with means smaller than 7.5% and 2.5% of the peak maximum, respectively. This identifies the rows in the image corresponding to the foreground plants. Third, the same column-cut and height-measurement algorithm used in the KAT4IA pipeline is applied to separate individual foreground plants and measure their heights. Figure 6A, C, and E shows examples of foreground-plant separation, and Fig. 6B, D, and F visualizes the height measurements for each of the target plants in those images. The proposed self-supervised CNN algorithm is able to recover most parts of the foreground plants with a low false-positive rate, and the height measurement algorithm provides reasonable estimates for the height of each target plant based on the foreground separation results.

**Fig. 7.**
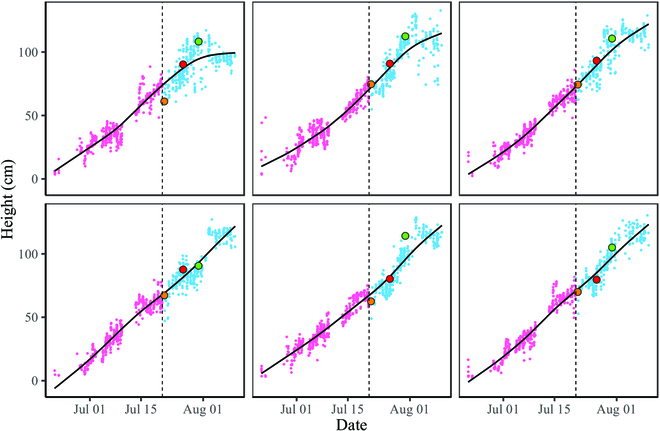
Growth curves are fitted to the height measurements for each plant in a set of images from one camera. The pink points are plant heights extracted from early-growth-stage images using the KAT4IA pipeline. The cyan-blue points are plant heights extracted from late-growth-stage images using our proposed method. Nondecreasing fitted growth curves are shown in black lines. The measured heights from the 3 images in Fig. 6 are highlighted with different colors. The orange dots indicate the heights from Fig. 6B, the red dots indicate the heights from Fig. 6D, and the green dots indicate the heights from Fig. 6F.

Using the extracted plant heights from all the plant images over the growth season, a growth curve for each plant in each row of the field can be fitted using nonparametric regression with a nondecreasing trend [24,25]. The fitted growth curves for the foreground plants shown in Fig. 6 are reported in Fig. 7. The pink dots and cyan-blue dots are the height measurements of the early- and late-growth-stage images, corresponding to the nonoverlapping plants and overlapping plants, respectively. The measured heights are also shown in Fig. 7 with different colors.

## Results

**Fig. 8.**
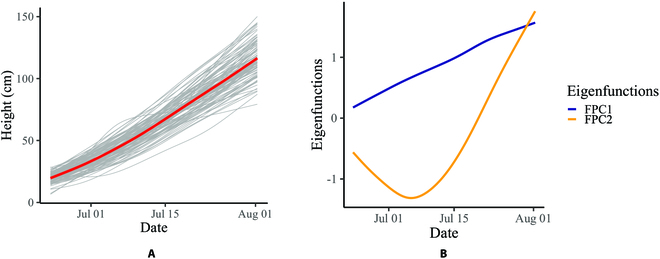
Growth curves were estimated for 103 genotypes. (A) Growth curve estimates for the 103 genotypes; the red curve represents the mean of the growth curve estimates. (B) First 2 estimated eigenfunctions.

In this section, we detail the results from applying our SS-CNN pipeline to the first replication of the 2017 dry field data. Specifically, we computed the median plant heights over the plant growing season for each of the 103 genotypes in the first replication and provided a growth curve estimation for each genotype. The growth curve estimates are shown in Fig. 8A, where the red curve represents the mean of the growth curve estimates. It is worth noting that most of the plants in the dry field were damaged by a storm in the beginning of August in 2017. Therefore, we decided to use the field images before August to fit growth curves. To investigate the effect of genotype on the plant-growth patterns, we applied an FPCA [21] to the 103 growth curve estimates. According to the Karhunen–Loève theorem, the growth curve for the *i*th genotype (*Y_i_*(*t*), *i* = 1, …, 103) can be well approximated by:

$$Y_{i}\left(t\right)=\mu \left(t\right)+\sum_{k=1}^{K}\xi_{\mathrm{ik}}\psi_{k}\left(t\right),$$

where *μ*(*t*) is the mean function, *ψ_k_*(*t*) is the *k*th eigenfunction, and *ξ_ik_* is the *k*th functional principal component (FPC) score for the *i*th genotype. The first 2 FPCs explain more than 95% of the total variance of the growth curve estimates. Note that we only measured plant heights before August, and most plants were still growing at that time. Therefore, the growth curves had not become flat. To fit a complete growth curve that flattens at the end, we can increase the model complexity by including more than 2 FPCs.

The first 2 eigenfunctions are plotted in Fig. 8B. The first eigenfunction controls the overall growth rate; a positive FPC score corresponding to the first eigenfunction results in a relatively higher growth rate than average. Meanwhile, the second eigenfunction controls the changes in growth rate over time; a positive FPC score corresponding to the second eigenfunction means that the growth rate increases over the plant growing season.

**Fig. 9.**
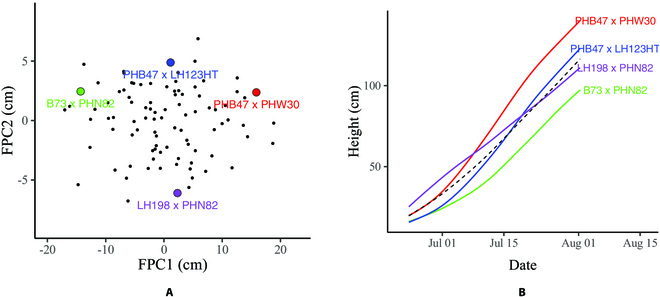
Growth curves correspond to a pair of FPC scores. (A) Scatterplot of the second FPC score versus the first FPC score for each genotype. (B) Growth curve estimates for 4 genotypes. Each growth curve corresponds to a pair of FPC scores marked using the same color in (A). The black dashed curve is the estimated mean growth curve.

Figure 9 provides a clearer explanation. Figure 9A shows a scatterplot of the second FPC score versus the first FPC score for each genotype. Genotypes *PHB47* × *PHW30* and *B73* × *PHN82* had similar second FPC scores; however, *PHB47* × *PHW30* had a positive first FPC score, whereas *B73* × *PHN82* had a negative first FPC score. Therefore, *PHB47* × *PHW30* had a higher overall growth rate than *B73* × *PHN82* (Fig. 9B). Genotypes *PHB47* × *LH123HT* and *LH198* × *PHN82* both had first FPC scores close to 0; however, the second FPC score of *PHB47* × *LH123HT* was >0, while the second FPC score of *LH198* × *PHN82* was <0. Therefore, the overall growth rates for both genotypes were close to the mean growth rate, but *PHB47* × *LH123HT* had a lower growth rate in the early growth stage and a higher growth rate in the late growth stage, whereas the growth rate of *LH198* × *PHN82* decreased over time.

## Discussion

Processing the image data and extracting plant features is one of the main problems in current phenotyping research and applications. Although human annotation for separating the target plants from background in some images is possible, this labeling process is usually tedious and time consuming. The proposed self-supervision approach uses computers to prepare the training data automatically. This allows a machine to efficiently measure plant traits from the images taken in fields that is adaptive to the lighting condition and environment change. Our results show that the proposed procedure can produce accurate and reliable measurements for plant height, and the following functional data analysis is able to reveal genotype effects on the plant growth curve. In the future, we will deploy the proposed analysis pipeline on an in-field imaging robot such that the robot can automatically extract plant traits and conduct statistical analysis in real time while working in the field.

In the proposed pipeline, we use sequential images to prepare the training data for the overlapped plants. The segmentation results rely on the similarity between the early-stage plants before overlapping and the late-stage plants after overlapping. If the color of the plant pixels from those 2 stages of images are quite different, the segmentation results would not be good. Fortunately, this is not the case in our image data analysis example. An alternative way to automatically create training data is to use the segmented plants from greenhouse images with a clean background and to overlay them on the images with the field background. This can create synthetic in-field plant images with known locations for the pixels of the foreground plants. Then, a CNN similar to the one used in the proposed pipeline or a U-net model [18] can be constructed for plant segmentation.

The validation of the extracted heights from images is an important question. However, we do not have manually measured plant heights from this experiment. So, we can not compare the extracted heights with the ground truth. Nevertheless, the extracted heights from the images should be accurate if the plant segmentation is accurate. The validity of the proposed segmentation procedure can be visually checked by comparing the segmented and original images, as shown in Fig. 6. In future experiments, we will collect manually measured plant heights over time as a validation dataset for the proposed procedure.

## Data Availability

The R code of the proposed pipeline, sample image data (including 6 field photos for training and 52 example field photos taken by one of our cameras for testing), and description are available on Github at https://github.com/xingcheg/Plant-Traits-Extraction-2023.
